# Gender Minority Stressors, Hopelessness, and Their Associations with Internalizing and Externalizing Mental Health Outcomes in a Hungarian Trans Adult Sample

**DOI:** 10.1007/s10508-025-03147-w

**Published:** 2025-05-07

**Authors:** Banu C. Ünsal, Zsolt Demetrovics, Melinda Reinhardt

**Affiliations:** 1 Doctoral School of Psychology, ELTE Eötvös Loránd University, Budapest, Hungary; 2 Institute of Psychology, ELTE Eötvös Loránd University, Budapest, Hungary; 3 Department of Child and Adolescent Psychiatry, Amsterdam University Medical Centers, Center of Expertise on Gender Dysphoria, Van der Boechorststraat 7, 1081 BT Amsterdam, The Netherlands; 4 Flinders University Institute for Mental Health and Wellbeing, College of Education, Psychology and Social Work, Flinders University, Bedford Park, SA Australia; 5 Centre of Excellence in Responsible Gaming, University of Gibraltar, Gibraltar, Gibraltar; 6 14th District Medical Center, Child and Adolescent Psychiatry, Budapest, Hungary

**Keywords:** Gender minority stress, Hopelessness, Depression, Anxiety, Substance use, Transgender

## Abstract

Although distal (i.e., discrimination, victimization, rejection, and nonaffirmation) and proximal (i.e., internalized transphobia, negative expectations, and identity nondisclosure) gender minority stressors are associated with internalizing (i.e., depression, anxiety, suicidality) and externalizing (i.e., substance use) mental health outcomes of trans individuals, how they are related to two distinct types of outcomes differs. General psychological processes (i.e., hopelessness) could explain the mechanisms behind the minority stressors-mental health association. Accordingly, this study aimed to test the complete gender minority stress model and the direct and indirect effects of minority stressors via hopelessness on mental health outcomes in trans individuals. Data were collected online from a convenience sample of 205 trans adults (18–74 years; *M* = 29.49, *SD* = 10.24), 72 (35.1%) of whom were trans men, 52 (25.4%) were trans women, and 81 (39.5%) were non-binary individuals. Results from structural equation modeling showed that distal stressors directly predicted mental health outcomes, except for depression. Internalized transphobia and negative expectations had positive indirect effects on depression, anxiety, and past-year and lifetime suicidality via hopelessness. Identity nondisclosure had negative indirect effects on depression, anxiety, and past-year suicidality through hopelessness. For substance use, hopelessness was not a significant mediator. Still, identity nondisclosure mediated distal stressors-substance use link. Findings suggest that hopelessness is a significant contributor to internalizing symptoms of trans individuals, making it a target for interventions to improve the mental health of trans people. The ameliorative impact of identity nondisclosure on both types of mental health outcomes should be considered and examined in further studies.

## Introduction

Trans individuals (i.e., whose gender identity, behavior, or expression doesn’t align with the sex they were assigned at birth) have consistently been found to experience symptoms of several internalizing (i.e., depression, anxiety, suicidality; Bränström et al., 2022; Connolly et al., 2016) and externalizing (i.e., substance use; Cotaina et al., 2022) mental health problems as compared to their cisgender counterparts. Although elevated levels of mental health problems experienced by trans people can partly be explained by the gender dysphoria (i.e., the distress about one’s body not matching one’s true gender identity) they experience (Dhejne et al., 2016), trans individuals are also situated in sociopolitical contexts characterized by anti-trans prejudice and stigma, which increases the mental health burdens among this population (Frost & Castro, 2024).

### Gender Minority Stress Model

One of the widely accepted and well-known explanations for these disproportionately higher levels of mental health problems in trans populations is proposed by the minority stress model (Frost & Meyer, 2023; Hendricks & Testa, 2012; Meyer, 2003; Testa et al., 2015). According to the gender minority stress model, trans individuals are exposed to socially based, unique, and chronic minority stressors originating from the power dynamics within societies. The model made a distinction between two different types of stressors, which are distal and proximal. Distal minority stressors are defined as real-life, prejudice-based, negative events, including gender-based discrimination, victimization, rejection, and non-affirmation of gender identity.

On the other hand, proximal stressors are defined as the subjective experiences of prejudice events that alter the stigmatized individual’s cognitive, emotional, or behavioral processes. Proximal stressors include internalized transphobia, negative expectations for future events, and identity nondisclosure. Internalized transphobia includes the process by which trans individuals internalize negative societal attitudes toward their identities and direct these attitudes toward themselves (Bockting, 2015). Negative expectations for future events describe the process through which trans individuals come to expect prejudice-based events to happen and become hypervigilant in their daily interactions with others (Hendricks & Testa, 2012). Finally, identity nondisclosure includes experiences of trans individuals during which they hide their trans identity from other people to avoid any prejudice-based events (Testa et al., 2015).

Previous literature provided consistent evidence that distal minority stressors were associated with depression and anxiety (Bockting et al., 2013; Budge et al., 2013; Puckett et al., 2023), suicidality (Guzman-Parra et al., 2023; Tebbe & Moradi, 2016; Testa et al., 2017), and substance use (Pellicane et al., 2025; Reisner et al., 2015; Wolford-Clevenger et al., 2021) among trans populations, demonstrating the robust negative impact of prejudice-based events on mental health. However, regarding proximal stressors, amendments to the minority stress model have been proposed because internalized transphobia and negative expectations for the future, but not identity nondisclosure, have consistently been found to be associated with negative mental health outcomes (Helsen et al., 2022; Mezza et al., 2024).

The impact of identity nondisclosure on mental health outcomes might further differ when conceptual differences between internalizing vs. externalizing mental health outcomes are considered. That’s to say, while previous literature showed that identity nondisclosure is associated with increased levels of internalizing mental health outcomes (Tebbe et al., 2022; Testa et al., 2017), it was associated with decreased levels of externalizing ones (Brennan et al., 2021). On the one hand, because individuals conceal their identity for fear of anticipated rejection and identity nondisclosure requires constant efforts to determine whether, when, how, and to whom the stigmatized identity can be disclosed, it depletes one’s cognitive and emotional capacity and leads to internalizing mental health problems (Pachankis, 2007). On the other hand, while individuals who disclose their identity might not experience the cognitive, emotional, and behavioral burden of concealment, their risk of developing substance use problems increases because they might become more involved with the trans community which is historically limited to be socialized in settings where substance use could be more common and a permissive and persistent norm (Hughes et al., 2016; Pachankis et al., 2020).

### Psychological Mediation Framework and Hopelessness as a Mediator

The minority stress model received a lot of attention from scholars and was shown to explain the mental health outcomes among trans populations. Since the model only focuses on group-specific processes, however, as an extension of the model (Frost & Meyer, 2023), the psychological mediation framework (Hatzenbuehler, 2009) was proposed which included both group-specific and general psychological risk factors. According to this framework, exposure to distal minority stressors causes the development of several cognitive (e.g., negative self-schemas), emotional (e.g., rumination), and social (e.g., social isolation) risk factors, which in turn leads to negative mental health outcomes. The framework also asserted that proximal stressors might further mediate the association of distal stressors with general psychological risk factors. The psychological mediation framework has been tested in trans populations previously by including rumination (Timmins et al., 2017), resilient coping (Logie et al., 2020), and shame (Scandurra et al., 2018) as mediators and was shown to be a valid framework for explaining the mental health of trans individuals.

One of the mediators of the minority stress-mental health link proposed by the psychological mediation framework was hopelessness. Hopelessness is defined as negative expectations regarding oneself and one’s future characterized by a lack of enthusiasm, and a motivational tendency to give up (Beck et al., 1974). Despite the theoretical overlap between hopelessness and negative expectations for the future as a proximal stressor, hopelessness not only involves expecting negative events in the future but further includes negative beliefs about one’s ability and capacity to cope with or change these events, which brings about a tendency to give up and feelings of pessimism (Hatzenbuehler, 2009).

Considering the serious barriers trans individuals experience in accessing higher education, employment, housing, and general and trans-related healthcare (European Union Agency for Fundamental Rights, 2020), hopelessness emerges as a fundamental consequence of minority stress and a potential contributor to poorer mental health outcomes among trans populations (Tebbe et al., 2022). Still, there is a scarcity of research investigating the role of hopelessness among trans populations. In one of these few studies (Nadal et al., 2014), feelings of hopelessness were reported as a common response to daily transphobic events. In another study (Tebbe et al., 2022), hopelessness was found to be a significant mediator of the link between discrimination, internalized transphobia, identity nondisclosure, and depressive and anxiety symptoms. However, negative expectations for future events were not included as a proximal stressor in this study and only internalizing mental health problems were examined. Thus, although these studies provide some evidence regarding the role of hopelessness in internalizing mental health problems, no single study, as far as we know, has extensively tested a model including all types of minority stressors, hopelessness, and internalizing and externalizing mental health outcomes simultaneously.

Furthermore, in Hungary, the human rights of trans individuals have been significantly changing lately including bans on legal gender recognition and parenting. (ILGA-Europe—The European Region of the International Lesbian, Gay, Bisexual, Trans and Intersex Association, 2018) Despite this, there is a scarcity of research examining the experiences of trans individuals in Hungary with most of the results coming from multi-national studies, reporting higher levels of discrimination (Falck & Bränström, 2023), victimization (Ünsal et al., 2023), and depression (Ünsal et al., 2024). Although these studies provide a general picture of the situation of trans individuals in Hungary, no single study, as far as we know, has extensively tested the gender minority stress model by including a variety of mental health outcomes among Hungarian trans individuals.

### The Current Study

**Fig. 1:**
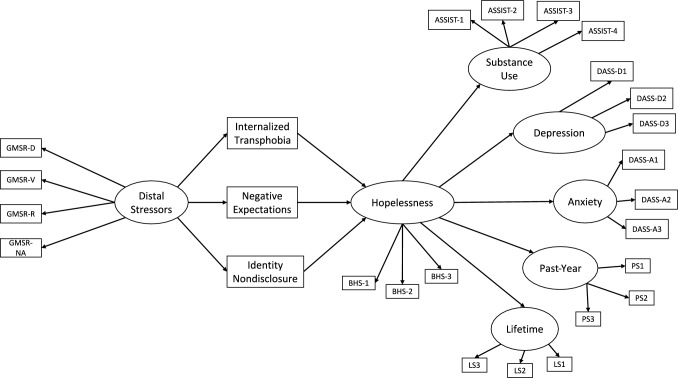
Proposed multiple sequential mediator model on the link between distal stressors and mental health outcomes. *Note.* GMSR-D, Gender minority stress and resilience measure discrimination subscale; GMSR-V, Gender minority stress and resilience measure victimization subscale; GMSR-R, Gender minority stress and resilience measure rejection subscale; GMSR-NA, Gender minority stress and resilience measure nonaffirmation subscale; BHS, Beck hopelessness scale; ASSIST, Alcohol, smoking, and substance involvement test; DASS-D, Depression, anxiety, stress scales depression subscale; DASS-A, Depression, anxiety, stress scales anxiety subscale; PS, Past-year suicidality; LS, Lifetime suicidality. Error terms and correlations between error terms were excluded for coherence

Based on the above, the purposes of this study were (1) to test the complete gender minority stress model, (2) to test the mediator role of hopelessness which was previously suggested as a significant and clinically relevant mediator in minority stress-mental health link, and (3) to examine whether and how the hypothesized associations would differ between internalizing vs. externalizing mental health outcomes. Accordingly, we hypothesized that (1) distal minority stressors would be positively associated with mental health problems, (2) internalized transphobia, negative expectations for future events, and identity nondisclosure would mediate the association of distal stressors with mental health outcomes, and (3) hopelessness would further mediate the relation between proximal stressors and mental health outcomes. More specifically, regarding the second set of hypotheses, we expected that distal stressors would positively predict internalized transphobia, negative expectations for future events, and identity nondisclosure, which in turn would be positively associated with internalizing mental health outcomes (i.e., depression, anxiety, and suicidality). However, we hypothesized that while internalized transphobia and negative expectations for future events would be *positively* associated with externalizing mental health outcomes (i.e., substance use), identity nondisclosure would be *negatively* associated. Regarding the third group of hypotheses, we expected that all three proximal stressors would be positively associated with hopelessness, which in turn would positively predict both internalizing and externalizing mental health outcomes (see Fig. 1).

## Method

### Participants

A total of 387 individuals started to fill out the online survey battery, 44 of whom did not answer any of the questions.^1^ Out of the remaining 343 individuals, 206 completed it until the end. However, one person was excluded due to being under the age of 18 years old. Thus, the final sample consisted of 205 trans individuals. We conducted chi-square tests on categorical sociodemographic variables and a *t*-test for the continuous age variable to compare the sample (*n* = 205) to those who did not complete the survey (*n* = 138). The only significant difference was found in terms of gender identity (*χ*^2^(2) = 6.85, *p* < .05), in a way that the proportion of non-binary individuals who completed the survey (*n* = 81, 67.8%) was higher compared to both trans men (*n* = 72, 59.8%) and trans women (*n* = 52, 50.5%). Also, the proportion of trans women who did not complete the survey (*n* = 51, 49.5%) was higher compared to trans men (*n* = 49, 40.2%) and non-binary individuals (*n* = 38, 32.2%).

Participant characteristics are provided in Table 1. Participants’ ages ranged between 18 and 74 (*M* = 29.49, *SD* = 10.24). The majority of participants were single (*n* = 127, 62%), living in an urban area (*n* = 46, 71.2%), were high school graduates or lower (*n* = 122, 59.5%), and were employed (*n* = 138, 67.3%). Regarding gender identity-related characteristics, 29 participants (14.1%) changed their legal gender whereas 65 of them (31.7%) changed their name legally. Seventy-nine of the participants (38.5%) reported that they had undergone at least one type of medical gender-affirming intervention. The most common medical gender-affirming intervention undergone by participants was hormone therapy (*n* = 77, 37.6%), followed by gender-affirming surgeries (*n* = 42, 20.5%), and hair removal (*n* = 33, 16.1%).

**Table 1:** Sociodemographic characteristics of the sample (*N* = 205)

	Trans men (*n* = 72, 35.1%)	Trans women (*n* = 52, 25.4%)	Non-binary/gender nonconforming (*n* = 81, 39.5%)
Age, *M *(*SD*)	27.93 (9.78)	33.23 (11.09)	28.47 (9.58)
Sexual orientation, *n* (%)			
Heterosexual	23 (31.9)	7 (13.5)	1 (1.2)
Lesbian/Gay	17 (23.6)	16 (30.8)	19 (23.5)
Bisexual	22 (30.6)	15 (28.8)	19 (23.5)
Pansexual	5 (6.9)	6 (11.5)	16 (19.8)
Asexual	1 (1.4)	6 (11.5)	12 (14.8)
Other	4 (5.6)	2 (3.8)	14 (17.3)
Minority status, *n* (%)			
Ethnic minority	3 (4.2)	5 (9.6)	4 (4.9)
Religious minority	6 (8.3)	4 (7.7)	12 (14.8)
Minority in terms of disability	10 (13.8)	2 (3.8)	16 (19.8)
Other minority	3 (4.2)	4 (7.7)	9 (11.1)
Relationship status, *n* (%)			
Single	42 (58.3)	30 (57.7)	55 (67.9)
In a relationship	30 (41.7)	22 (42.3)	26 (32.1)
Place of residence, *n* (%)			
Urban	47 (65.3)	37 (71.2)	62 (76.6)
Rural	25 (34.7)	15 (28.8)	19 (23.4)
Education level, *n* (%)			
Highschool or lower	45 (62.5)	25 (48.1)	52 (64.2)
University degree or higher	27 (37.5)	27 (51.9)	29 (35.8)
Employment status, *n* (%)			
Employed	46 (63.9)	39 (75.0)	53 (65.4)
Unemployed	26 (36.1)	13 (25.0)	28 (34.6)
Legal gender change, *n* (%)			
Yes	12 (16.7)	12 (23.1)	5 (6.2)
No	60 (83.3)	40 (76.9)	76 (93.8)
Gender-affirming intervention, *n* (%)			
Yes	35 (48.6)	28 (53.8)	16 (19.8)
No, but I want to	26 (36.1)	12 (23.1)	37 (45.7)
No, and I don’t want	11 (15.3)	12 (23.1)	28 (34.6)
Type of intervention, *n* (%)			
Puberty blockers	0 (0)	7 (13.5)	1(1.2)
Hormone therapy	35 (48.6)	26 (50.0)	16 (19.8)
Surgery	26 (36.1)	10 (19.2)	6 (7.4)
Hair removal	0 (0)	27 (51.9)	6 (7.4)
Voice training/surgery	2 (2.8)	15 (28.8)	1 (1.2)
Other	0 (0)	2 (3.8)	0 (0)

Participants reported their sexual orientation based on their self-identified gender

### Procedure

For the current study, we used the baseline data from a longitudinal study examining the relations between gender minority stressors, general psychological risk factors, and mental health outcomes in a Hungarian sample of trans adults. Data for the baseline were collected online between September and November 2023. The questionnaire battery was uploaded into Qualtrics, and the study was advertised on social media accounts of several Hungarian transgender non-governmental organizations, Facebook groups for trans individuals in Hungary, and the webpage of the LGBTIQ Section of the Hungarian Psychological Association. Being at least 18 years of age, speaking Hungarian to complete the survey, and self-identifying with a trans identity were required to be included in the study. Participation was completely anonymous and voluntary. All participants were provided with an informed consent form at the beginning of the survey. Participation in the study took approximately 30 min and a list of mental and gender-affirmation-related health resources that participants could benefit from in Hungary were provided at the end of the survey.

### Measures

#### Gender Identity

Individuals reported how they define their current gender identity with the following response options: “woman/feminine,” “man/masculine,” “non-binary,” and “other.” They also reported if they identified as a trans person with the response options: “Yes, I am a trans woman,” “Yes, I am a trans man,” “Yes, I am a non-binary trans person,” “Yes, I am a trans person with the following identity,” and “No, I am not a trans person.” Based on similar previous studies (e.g., Helsen et al., 2022; Testa et al., 2015), individuals were grouped into three categories (i.e., trans women, trans men, and non-binary) based on their answers to gender identity and trans status questions.

#### Demographic Information Form

Participants’ basic sociodemographic and gender identity-related information was obtained with a demographic information form prepared by the authors. The first part of the form included questions on age, sexual orientation, place of residence, relationship status, education level, employment status, and ethnic, religious, disability, and other minority statuses. More specifically, for sexual orientation, individuals answered how they define their sexual orientation based on their self-identified gender with the following response options: “heterosexual/straight,” “gay/lesbian,” “bisexual,” “pansexual,” “asexual,” and “other: please identify.”

The second part of the form asked questions on legal gender change, name change, and gender-affirming interventions, including puberty blockers, hormone treatment, gender-affirming surgeries, hair removal, voice training, and other types of interventions (e.g., hair transplant, thyroid chondroplasty).

#### Distal Stressors

Gender Minority Stress and Resilience Measure (GMSR; Testa et al., 2015), which is a 58-item self-report scale with nine subscales that measure minority stress and resilience experiences of trans people, was used to measure distal stressors. More specifically, we used (1) five-item gender-related discrimination (e.g., I have experienced difficulty getting identity documents that match my gender identity), (2) seven-item gender-related victimization (e.g., I have had my personal property damaged because of my gender identity or expression), (3) six-item gender-related rejection (e.g., I have been rejected or distanced from friends because of my gender identity or expression), and (4) six-item non-affirmation of gender identity (e.g., I have difficulty being perceived as my gender) subscales of the GMSR. The response options to the items of the gender-related discrimination, victimization, and rejection subscales included “Yes, before age 18,” “Yes, after age 18,” “Yes, in the past year,” and “never.” Participants selected all items that applied to them. The responses were coded as 0 if “never,” and as 1 for “yes” at any point are selected. For the non-affirmation of gender identity subscale, responses were on a 5-point scale ranging from 0 (*strongly disagree*) to 4 (*strongly agree*). The total score for each subscale was calculated by summing up the responses given to all items of their respective subscale. The Cronbach’s alpha reliability scores for the gender-related discrimination, victimization, rejection, and non-affirmation of gender identity were .68, .70, .62, and .89, respectively for the current sample.

#### Internalized Transphobia

Internalized transphobia was assessed by using the eight-item Internalized Transphobia subscale of the GMSR (Testa et al., 2015). An example item of this subscale is “I resent my gender identity or expression.” The items were rated on a 5-point scale with responses ranging from 0 (*strongly disagree*) to 4 (*strongly agree*). A total score of internalized transphobia was calculated by summing up the responses given to the eight items. Higher scores represent higher internalized transphobia levels. For the current sample, the Cronbach’s alpha reliability score was .83.

#### Negative Expectations for Future

Negative Expectations for the Future were assessed by using the nine-item negative expectations for the future subscale of the GMSR (Testa et al., 2015). An example item of this subscale is “If I express my gender identity, most people would look down on me.” The items were rated on a 5-point scale with responses ranging from 0 (*strongly disagree*) to 4 (*strongly agree*). A total score of negative expectations for the future was calculated by summing up the responses given to the nine items. Higher scores represent more negative expectations for the future. For the current sample, the Cronbach’s alpha reliability score was .89.

#### Identity Nondisclosure

Identity nondisclosure was assessed by using the five-item Identity Nondisclosure subscale of the GMSR (Testa et al., 2015). An example item of this subscale is “Because I don’t want others to know my gender identity, I modify my way of speaking.” The items were rated on a 5-point scale with responses ranging from 0 (*strongly disagree*) to 4 (*strongly agree*). A total score of identity nondisclosure was calculated by summing up the responses given to the five items. Higher scores represent higher levels of identity nondisclosure. For the current sample, the Cronbach’s alpha reliability score was .82.

#### Hopelessness

Hopelessness was assessed by using the short version of the Beck Hopelessness Scale (BHS; Perczel Forintos et al., 2013). The short version of the BHS consists of three dichotomous true/false items. An example item is “My future seems dark to me*.*” Total hopelessness score is calculated by responses given to three items. Higher scores represent higher hopelessness levels. Cronbach’s alpha reliability score for the current sample was .74.

#### Depression and Anxiety

Nine-item Depression, Anxiety, and Stress Scales (DASS-9; Yusoff, 2013), which is an empirically derived shortened version of the original 21-item scale (DASS-21; Lovibond et al., 1995; Szabó, 2010) was used to assess depression and anxiety. DASS-9 measures symptoms of depression, anxiety, and stress in the last week with three three-item subscales. The responses are on a 4-point scale with response options ranging from 0 (*did not apply to me at all*) to 3 (*applied to me very much, or most of the time*). An example item of depression is “I was unable to become enthusiastic about anything.” An example item of anxiety is “I felt I was close to panic.” Total depression and anxiety scores are calculated by summing up responses given to the items, with higher scores representing higher symptoms of depression and anxiety. DASS-21 demonstrated good reliability and validity scores and measurement invariance between cisgender and TGNC individuals (Lindley & Bauerband, 2023). The nine-item version was used to decrease the burden on participants in filling out the survey. Cronbach’s alpha reliability scores for both the depression and anxiety subscales were .81 for the current sample.

#### Suicidality

Suicidality was assessed with three items asking participants how frequently they have suicidal thoughts, ideation, and plans. The participants answered the items for their whole life (i.e., lifetime suicidality) and for the last year (i.e., past-year suicidality). The response options included “*never* [coded as 1],” “*sometimes* [coded as 2],” “*often* [coded as 3],” and “*always* [coded as 4].” Total lifetime and past-year suicidality scores were calculated by summing up responses given to three items, with higher scores representing higher suicidality. Cronbach’s alpha reliability scores for the lifetime and past-year suicidality were .89 and .88, respectively.

#### Substance Use

Substance use was assessed with the 11-item Alcohol, Smoking, and Substance Involvement Screening Test (ASSIST-11; Lee et al., 2023), which measures the frequency of use of several substances in the last three months and has been found to have measurement invariance between cisgender and trans individuals. We included five items from ASSIST-11 in the current study based on their higher usage prevalence estimates (Chang et al., 2022), which were tobacco, alcohol, cannabis, prescribed drugs, and others. The response options included “*never,*” “*once or twice,*” “*monthly,*” “*weekly,*” and “*daily or almost daily.*” Cronbach’s alpha reliability score in the current sample was .50.

### Statistical Analyses

Descriptive statistics and the bivariate Pearson’s correlation coefficients between the study variables were calculated by SPSS version 29. Structural equation modeling (SEM) was used for model testing by MPlus version 8. The amount of missing data ranged from 0.5% (i.e., substance use) to 2% (i.e., negative expectations for the future). Missing values were treated with full information maximum likelihood. We allowed error terms for manifest variables to correlate if we had theoretical justifications and doing so resulted in a significant improvement in model fit.

We started our analyses with a model that only included distal stressors and mental health outcomes. This model tested the direct path from distal stressors to mental health outcomes. We then added internalized transphobia, negative expectations for the future, and identity nondisclosure into the model to test the indirect paths from distal stressors to mental health outcomes via these three variables. Maximum likelihood with robust standard errors (MLR) was used for the first two models. In the third model, we added hopelessness and tested the indirect paths from all variables to the mental health outcomes through hopelessness. Weighted least square estimation (WLSMV) was used for the third model given that hopelessness items were categorical. Finally, following previous similar research (Timmins et al., 2017), we also included theoretically relevant sociodemographic variables as control variables, including age, education level, relationship status, place of residence, and gender-affirming interventions.

Indirect effects were considered to be significant if the 95% confidence intervals (CI) did not include zero. We followed previous general recommendations to assess the goodness of fit of each model, which were chi-square/degrees of freedom (χ^2^/df), root mean square error of approximation (RMSEA; < .05), standardized root mean square residual (SRMR; < .10), comparative fit index (CFI; > .95 or > .90), and Tucker-Lewis index (TLI; > .95 or > .90).

## Results

### Descriptive Statistics

Descriptive statistics and bivariate Pearson’s correlation coefficients between the variables are presented in Table 2. All variables were found to be relatively normally distributed and the Pearson’s correlation coefficients were significant in the expected directions, except for substance use, which had higher skewness and kurtosis scores and was correlated only with gender-related rejection, victimization, and lifetime suicidality.

**Table 2:** Descriptive statistics and correlations between the study variables

Variable	1	2	3	4	5	6	7	8	9	10	11	12	13	*M (SD)*	Skew	Kurt
1. Discrimination	–													2.23 (1.61)	.17	− 1.05
2. Rejection	.56**	–												2.64 (1.66)	− .04	− .86
3. Victimization	.44**	.46**	–											1.92 (1.57)	.62	− .51
4. Nonaffirmation	.30**	.24**	.13	–										13.59 (7.23)	− .41	− 1.00
5. IT	.17*	.29**	.23**	.38**	–									14.27 (8.03)	.19	.74
6. NE	.33**	.31**	.26**	.41**	.48**	–								18.64 (8.47)	− .21	− .52
7. Nondisclosure	.42**	.38**	.22**	.23**	.41**	.51**	–							9.37 (5.77)	.05	− .95
8. Hopelessness	.09	.07	.08	.39**	.40**	.38**	.17*	–						1.30 (1.19)	.22	− 1.48
9. Depression	.14*	–.02	.05	.35**	.38**	.36**	.23**	.60**	–					3.92 (2.62)	.24	− .95
10. Anxiety	.31**	.17**	.18**	.36**	.33**	.35**	.35**	.42**	.45**	–				2.86 (2.71)	.79	− .42
11. Lifetime	.27**	.19**	.19**	.17*	.28**	.26**	.24**	.28**	.36**	.30**	–			6.61 (2.44)	.49	− .45
12. Past-year	.20**	.13	.21**	.18**	.31**	.29**	.18*	.36**	.49**	.44**	.66**	–		5.11 (2.37)	1.31	1.08
13. SU	.10	.19**	.17*	.03	− .03	− .01	.02	.10	− .06	.09	.22**	.12	–	8.54 (3.38)	1.41	2.93

IT, Internalized transphobia; NE, Negative expectations for future; Lifetime, Lifetime suicidality, Past-year, Past-year suicidality; SU, Substance use; Skew., Skewness; Kurt., Kurtosis. *p < .05, **p < .01

### Predictor Role of Distal Stressors on Mental Health Outcomes

The first SEM model testing the direct path from distal stressors to mental health outcomes did not have an acceptable fit: χ^2^(174) = 404.76, *p* < .001; RMSEA = .08, 90% CI [.07–.09], SRMR = .08, CFI = .86, TLI = .83. Investigation of modification indices and factor loadings demonstrated that (1) item about prescribed drug use did not significantly load onto the substance use variable and (2) lifetime and past-year suicidality items had significant cross-loadings. Thus, the item regarding prescribed drug use was excluded from the analyses, and error terms between items of the lifetime and past-year suicidality were allowed to correlate, given that the items measure the same constructs in different time frames. After these modifications, the model significantly improved: χ^2^(148) = 229.85, *p* < .001; RMSEA = .05, 90% CI [.04–.07]; SRMR = .05, CFI = .95, TLI = .94, and subsequent models were based on these modifications. Distal stressors significantly and positively predicted anxiety (β = .23, *p* < .001), lifetime (β = .21, *p* < .001) and past-year suicidality (β = .19, *p* < .01), and substance use (β = .16, *p* < .01). However, the association of distal stressors with depression was nonsignificant (β = .07, *p* = .11).

### Mediator Roles of Proximal Stressors

In a second model, proximal stressors (i.e., internalized transphobia, negative expectations for the future, and identity nondisclosure) were included, and indirect paths from distal stressors to mental health outcomes via these variables were tested. The model fit slightly improved: χ^2^(188) = 282.02, *p* < .001; RMSEA = .05, 90% CI [.04–.06]; SRMR = .05, CFI = .95, TLI = .94. Partially supporting our hypotheses, significant mediator roles of proximal stressors were found between distal stressors and mental health outcomes. Notably, identity nondisclosure mediated the association of distal stressors with substance use (β = − .15, *p* < .05). Regarding depression, both internalized transphobia (β = .11, *p* < .01) and negative expectations for the future (β = .15, *p* < .01) were significant mediators. For past-year suicidality, internalized transphobia (β = .07, *p* < .05) was a significant mediator. However, regarding anxiety and lifetime suicidality, no mediation effect was found.

### Mediator Role of Hopelessness

**Fig. 2:**
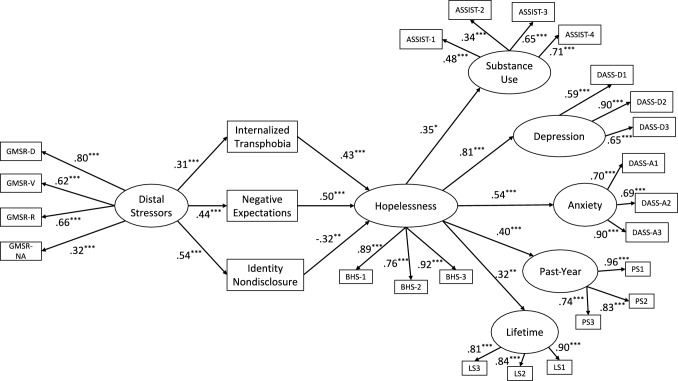
Final model of the indirect association between distal stressors and mental health outcomes via proximal stressors and hopelessness. *Note.* GMSR-D, Gender minority stress and resilience measure discrimination subscale; GMSR-V, Gender minority stress and resilience measure victimization subscale; GMSR-R, Gender minority stress and resilience measure rejection subscale; GMSR-NA, Gender minority stress and resilience measure nonaffirmation subscale; BHS, Beck hopelessness scale; ASSIST, Alcohol, smoking, and substance involvement test; DASS-D, Depression, anxiety, stress scales depression subscale; DASS-A, Depression, anxiety, stress scales anxiety subscale; PS, Past-year suicidality; LS, Lifetime suicidality. Standardized regression coefficients are presented. Error terms and correlations between error terms were excluded for coherence. ***p < .001, **p < .01, *p < .05

In a third model, hopelessness was included, and indirect paths from three proximal stressors to mental health outcomes via hopelessness were tested. The model fit improved significantly and the model had the best fit: χ^2^(249) = 281.36, *p* = .08; RMSEA = .03, 90% CI [.00–.04]; SRMR = .05, CFI = .97, TLI = .96. Including covariates did not change the overall pattern of results. Thus, for the principle of parsimony, they were excluded from the final model. The results are visually depicted in Fig. 2. Partially supporting our hypotheses, significant mediator roles of hopelessness were found between three proximal stressors and mental health outcomes (see Table 3). For substance use, hopelessness was not a significant mediator for any indirect paths. Still, the direct effect of distal stressors on substance use was significant after controlling for all other variables (β = .46, *p* < .001).

**Table 3:** Results of the multiple sequential mediation model

	*β* (95% CI)
Common paths	
DS ➔ IT	**.31 (.16, .46)**
DS ➔ NE	**.44 (.29, .59)**
DS ➔ ND	**.54 (.40, .68)**
IT ➔ Hopelessness	**.43 (.26, .60)**
NE ➔ Hopelessness	**.50 (.31, .69)**
ND ➔ Hopelessness	− **.32 (**− **.55, **− **.09)**

	Substance use	Depression	Anxiety	Past-Year	Lifetime
Outcome-specific paths					
Hopelessness ➔ Outcome	**.35 (.07, .62)**				
		**.81 (.58, 1.04)**			
			**.54 (.31, .77)**		
				**.40 (.18, .61)**	
					**.32 (.10, .54)**
Indirect effects via hopelessness					
IT	.05 (− .01, .10)	**.11 (.03, .19)**	**.07 (.01, .13)**	**.05 (.01, .10)**	**.04 (.01, .08)**
NE	.08 (− .01, .15)	**.18 (.06, .30)**	**.12 (.03, .21)**	**.09 (.02, .16)**	**.07 (.01, .13)**
ND	− .06 (− .13, .01)	− **.14 (**− **.26, **− **.01)**	− **.09 (**− **.18, **− **.01)**	− **.07 (**− **.13, **− **.01)**	− .06 (− .11, .01)
Direct effect	**.46 (.25, .67)**	− .17 (− .36, .02)	.15 (− .05, .35)	.19 (− .01, .39)	**.23 (.03, .43)**

DS, Distal stressors; IT, Internalized transphobia; NE, Negative expectations for the future; ND, Identity nondisclosure; Past-year, Past-year suicidality; Lifetime, Lifetime suicidality; CI, Confidence interval. Standardized regression coefficients are reported. Significant effects are boldfaced for readability

For depression, indirect effects of internalized transphobia (β = .11, *p* < .01) and negative expectations for the future (β = .18, *p* < .01), via hopelessness were positive and significant. On the other hand, the indirect effect of identity nondisclosure (β = − .14, *p* < .05) via hopelessness was negative and significant. More specifically, higher identity nondisclosure predicted lower levels of hopelessness (β = − .32, *p* < .01), which in turn predicted lower levels of depression (β = .81, *p* < .001). Also, the direct effect of distal stressors on depression was nonsignificant (β = − .17, *p* = .08). Regarding anxiety, similar to depression, positive and significant indirect effects of internalized transphobia (β = .07, *p* < .05) and negative expectations for the future (β = .12, *p* < .01) and a negative and significant indirect effect of identity nondisclosure (β = − .09, *p* < .05) via hopelessness were found. Also, the direct effect of distal stressors on anxiety became nonsignificant (β = .15, *p* = .14).

Regarding past-year suicidality, similar to depression and anxiety, positive and significant indirect effects of internalized transphobia (β = .05, *p* < .05) and negative expectations for the future (β = .09, *p* < .05) and a negative and significant indirect effect of identity nondisclosure (β = − .07, *p* < .05) via hopelessness were found. Also, the direct effect of distal stressors on past-year suicidality became nonsignificant (β = .19, *p* = .05). Regarding lifetime suicidality, positive and significant indirect effects of internalized transphobia (β = .04, *p* < .05) and negative expectations for the future (β = .07, *p* < .05) were found. However, the indirect effect of identity nondisclosure via hopelessness was nonsignificant (β = − .06, *p* = .06) and the direct effect of distal stressors on lifetime suicidality remained significant (β = .23, *p* < .05).

The results are summarized in Table 3 with confidence intervals and specific path coefficients. All in all, this model explained 23% of the variance in substance use, 70% in depression, 42% in anxiety, 24% in past-year suicidality, 23% in lifetime suicidality, and 41% in hopelessness.

## Discussion

The current study aimed to test the complete minority stress model and to investigate the mediator role of hopelessness in the association of minority stressors with both internalizing and externalizing mental health outcomes in a Hungarian trans sample of adults. Overall, our findings support most of the main tenets of the minority stress model (Hendricks & Testa, 2012; Meyer, 2003) and the psychological mediation framework (Hatzenbuehler, 2009). First, we found evidence that distal minority stressors significantly predicted higher levels of mental health problems, supporting previous literature demonstrating the negative impacts of prejudice-based events on mental health (Nicholas & Bresin, 2024; Puckett et al., 2023; Tebbe & Moradi, 2016; Wolford-Clevenger et al., 2021).

One exception to that was the nonsignificant association of distal stressors with depression. Still, in later models with proximal stressors and hopelessness as mediators, we found significant indirect effects on depression. More specifically, we found that internalized transphobia and negative expectations for the future mediated the distal stressors-depression association, which is in line with the results from several previous studies (Bockting et al., 2013; Tebbe & Moradi, 2016). As stated in the introduction section, because internalized transphobia includes negative evaluations of oneself for being trans and negative expectations for the future comprise cognitions of expecting prejudice-based treatment from others, the cognitive-evaluative dimensions of these stressors might account for the indirect effects on depression, which also has cognitive and evaluative aspects (Gotlib & Joormann, 2010). Thus, it can be asserted that the subjective experience and appraisal of distal stressors might be more relevant in depressive symptomatology among trans populations.

Furthermore, in line with previous findings (Tebbe et al., 2022), we found that hopelessness was a significant mediator in the links between three types of proximal stressors and depression, with indirect effects of internalized transphobia and negative expectations for the future being positive and identity nondisclosure being negative. Given that hopelessness also involves negative cognitions regarding one’s future, these results further emphasize the significance of cognitive processes in the development of depressive symptoms among trans populations. The indirect effect of identity nondisclosure via hopelessness being significant but negative indicates that identity nondisclosure is differentially linked to depression. Several lines of previous research also showed that identity nondisclosure predicted lower levels of depression in trans populations (Timmins et al., 2017; Ünsal et al., 2024). One explanation for this is that concealing one’s identity decreases one’s exposure as a minority to others, which in turn decreases their exposure to distal stressors and negative expectations for the future. Considering the recent sociopolitical changes regarding the human rights of trans individuals in Hungary, identity nondisclosure might have a protective role against prejudice-based events in our sample, which was also shown by previous studies on structural stigma and identity concealment (Bränström & Pachankis, 2021).

Regarding the mediator roles of three proximal stressors on the distal stressors-substance use link, we found evidence that only identity nondisclosure significantly mediated this association. This result is in line with previous evidence demonstrating that concealing one’s identity was associated with lower levels of substance use problems (Peacock et al., 2015), as it potentially limits the socialization of trans people in settings with more permissive substance use norms (Cotaina et al., 2022; Pachankis et al., 2020). Internalized transphobia and negative expectations for the future were not significant mediators. Moreover, hopelessness was not a significant mediator between any proximal stressors and substance use, and the direct effect of distal stressors was still significant even after controlling for these variables. These findings indicate that substance use is both directly associated with negative interpersonal events such as rejection and indirectly via identity nondisclosure, which is the most interpersonal type of proximal minority stressors. Thus, it is plausible to state that interpersonal processes play a distinctive role in the development of substance use problems among trans populations, which were previously also suggested to be a significant determinant in the general population (Leach & Kranzler, 2013).

With regards to anxiety, although proximal stressors were not significant mediators of distal stressors-anxiety association, they had significant indirect effects via hopelessness, and the direct effect of distal stressors became nonsignificant. Although proximal stressors not being significant mediators contradicts the minority stress model (Hendricks & Testa, 2012; Meyer, 2003), the significant mediator role of hopelessness supports the psychological mediation framework (Hatzenbuehler, 2009), postulating that general psychological processes would mediate the minority stress-mental health association. This finding indicates the importance of hopelessness in the anxiety symptoms of trans individuals. Since items measuring anxiety in the current study captured physiological hyperarousal symptoms of anxiety (e.g., I experienced trembling), our findings suggest that hopeless cognitions regarding one’s future are associated with elevated levels of such symptoms. Previous literature (Arnau, 2017) also suggested that anxious apprehension, which is triggered by perceiving oneself as unable to control, prevent, or change an actual or anticipated negative event, leads to a feedback loop during which individuals focus on their perceived inadequacy, which activates their hopeless cognitions, which in turn increases their arousal (Corrigan & Schutte, 2023). Thus, anxious apprehension and hopeless appraisal of minority stressors might further increase the impact of minority stressors on the development and maintenance of anxiety symptomatology among trans individuals.

Regarding past-year suicidality, although only internalized transphobia was a significant mediator in the association between distal stressors and past-year suicidality, when hopelessness was included, all three proximal stressors had significant indirect effects via hopelessness and the direct effect of distal stressors became nonsignificant. These findings highlight the importance of hopelessness in recent suicidality symptoms among trans populations. Not surprisingly, hopelessness has been studied extensively and found to be a significant predictor of suicidality in the general population (Ribeiro et al., 2018). Because hopeless cognitions comprise beliefs that one’s conditions will not change in the future, this leaves people with fewer reasons to live and eventually leads to thoughts, ideas, and plans to terminate their own lives (Bagge et al., 2014). Internalized transphobia being the only significant mediator further emphasizes the relevance of negative self-evaluations in recent suicidality symptoms in trans individuals. On the other hand, similar to depression and anxiety, the indirect effect of identity nondisclosure via hopelessness being negative further underlines the potential protective impact of identity nondisclosure on mental health, including past-year suicidality.

Finally, for lifetime suicidality, although none of the proximal stressors were significant mediators, when hopelessness was included, internalized transphobia and negative expectations for the future had significant indirect effects via hopelessness. Still, the direct effect of distal stressors on lifetime suicidality remained significant. One explanation for this might be that both lifetime suicidality and distal stressors capture and assess one’s whole life, unlike other study variables which assessed the current situation of participants. Identity nondisclosure not being a significant mediator for lifetime suicidality demonstrates that currently not disclosing one’s trans identity emerges as a protective factor only for more recent symptoms of suicidality (i.e., past year) but not for the lifetime presence of such symptoms. This result is not surprising from a developmental perspective because trans individuals might be more likely to disclose their identities during adolescence and early adulthood years (Restar et al., 2019; Tatum et al., 2020), for which we couldn’t assess their identity nondisclosure experiences. Overall, these results suggest that distal and proximal minority stressors have different impacts on suicidality symptoms via hopelessness based on their temporal proximity.

### Clinical and Policy Implications

Our findings have several clinical and policy implications regarding the mental health of trans individuals. First, because distal stressors emerged as significant predictors of mental health outcomes, public policies tackling gender-related discrimination, rejection, and victimization should be developed and implemented to improve the mental health of trans people. Secondly, as shown by the findings, the indirect effect of identity nondisclosure on both externalizing and internalizing mental health outcomes was negative, meaning that identity nondisclosure could be protective in both types of mental health outcomes. Thus, clinicians working with trans individuals should integrate identity nondisclosure into their practice and should support the identity management processes of their clients by benefitting from the decision-making model of identity (non)disclosure (Chaudoir & Fisher, 2010), which asserts that disclosing one’s identity is a continuous process during which individuals constantly evaluate their social settings and the pros and cons of their disclosure.

Moreover, despite the lower significance level, since our results demonstrated that only minority stress processes with more interpersonal aspects predicted substance use, these aspects should be considered in clinical practice. A potential way of doing this is to provide these individuals with more adaptive skills to cope with negative interpersonal interactions (Davies et al., 2024). Another way would be to create alternative social settings with less permissive substance use norms (i.e., sober queer places), such as queer coffee shops, bookstores, and centers for cultural events and to encourage trans people to socialize in such settings (Connolly et al., 2024), which would decrease their exposure to substance-saturated environments (Felner et al., 2020). Still, it is important to note that the proportion of variance explained in substance use was relatively lower in the current study. Thus, the clinical implications mentioned here should be applied cautiously, and other potential variables that could explain a higher variance in substance use should be considered in clinical practice. Previous literature identified emotion regulation, rumination, and substance expectancies as factors contributing to substance use problems (Hatzenbuehler, 2009), thus these factors could be considered in clinical practice with trans individuals having substance use problems.

Furthermore, hopelessness was found to be a significant mediator between minority stressors and internalizing mental health outcomes. Thus, hopelessness should be carefully assessed and interventions targeting it should be integrated into clinical practice. Regarding depression and suicidality, the association of negative cognitions regarding oneself and anticipated negative treatment from others with hopelessness should be addressed. Regarding anxiety, the potential contribution of the abovementioned anxious apprehension and the hopeless feedback loop (Corrigan & Schutte, 2023) should be considered and tackled.

Cognitive-behavioral therapy guidelines could provide a framework to tackle hopelessness in clinical practice (Beck, 2020). More specifically, cognitive restructuring that aims to modify distorted cognitions of depicting the future as inevitable and unchangeable could be implemented to decrease hopelessness (Marchetti et al., 2023). Automatic negative thoughts related to negative inferences about one’s ability to cope with or change one’s future should also be tackled, which will increase the individuals’ sense of control for actual or anticipated events in the future (Marchetti et al., 2023).

The Best Possible Self (King, 2001) is another psychotherapeutic technique during which individuals are required to envision themselves in an imaginary future in which everything in their lives turns out the way they wish. This technique allows the clinician and the client to directly identify the dismal expectations about the client’s future, tackle the potential roadblocks that could impede their goal attainment, and help develop more realistic future goals. All these specific techniques should be integrated into a trans-affirmative cognitive-behavioral therapy approach (Austin & Craig, 2019) to ensure that the unique challenges and needs of trans individuals including distal and proximal stressors and their associations with hopelessness are targeted.

Last but not least, the adverse mental health outcomes experienced by trans people mostly originate from anti-trans social and political structures (White Hughto et al., 2015). Thus, to decrease the one-sided burden on trans individuals in improving their mental health, all individual-level interventions should be accompanied and sustained by structural-level changes that ensure the protection of their fundamental human rights.

### Limitations and Directions for Future Studies

Despite providing important insights into the mental health of trans individuals, our study has several limitations. First, the cross-sectional design of the study prevents us from inferring any causal associations between the study variables. Longitudinal and/or experimental studies should be conducted to test the cause-effect relations among minority stressors, hopelessness, and mental health outcomes. Second, although we recruited one of the biggest samples of the Hungarian trans population, our sample was not representative and the data were collected by self-report measures, which limits the generalizability of our findings and increases self-report and social desirability biases. Future studies with more heterogeneous and representative samples and different data collection methods are needed in that regard. Moreover, although ASSIST-11 has previously been shown to demonstrate a lower-than-expected reliability score and higher skewness and kurtosis scores since the measure assesses the use of several different substances with a single broad substance use factor (Lee et al., 2023), these scores might still have attenuated our results regarding substance use. Similarly, distal stressors subscales of the GMSR had relatively lower reliability scores in our study, which was also reported previously (Lindley et al., 2024). These lower reliability scores might have impacted the overall pattern and magnitude of results. Thus, future studies should develop measurement tools that can reliably assess distal stressors among trans populations. In a similar vein, although the identity nondisclosure subscale of the GMSR is one of the widely used tools to measure identity nondisclosure experiences of trans people, a recent scoping review (Osmetti et al., 2025) of the current literature suggested that the subscale assesses effortful concealment behaviors and shows weaker correlations with mental health outcomes compared to other two proximal stressors. This might not only reflect the simultaneous protective and distressing impacts of identity nondisclosure on mental health but also indicate a flaw in the identity nondisclosure subscale of GMSR (Jones et al., 2024). Thus, the conclusions about identity nondisclosure in the current study should be interpreted cautiously, and future studies should develop novel measures to capture the complex nature of trans identity nondisclosure.

Additionally, although we could explain higher variance in depression, anxiety, and hopelessness, the proportions of variance accounted for in suicidality and substance use were relatively lower. Thus, future studies should include other potential mediator variables (e.g., substance expectancies, social isolation/loneliness, rumination) to better understand the mechanisms of these mental health outcomes. This is especially relevant for substance use where identity nondisclosure is included as a proxy for community involvement to test the assumption that those who disclose their identity more will be more involved with the trans community. Thus, future studies directly measuring community involvement are needed to provide a clearer picture of the associations between these variables. Similarly, because only minority stressors were included in this study, we were not able to identify resilience-related factors that could potentially ameliorate the mental health outcomes of trans individuals. Thus, future studies should also involve resilience-related factors (e.g., gender euphoria, and community connectedness) to examine the role of protective factors on mental health. Finally, because the negative association of identity nondisclosure with mental health outcomes contradicts the minority stress model and the psychological mediation framework, identity nondisclosure experiences of trans individuals should be examined and elaborated thoroughly in future studies to determine whether adjustments to this construct are needed in these theoretical frameworks.

### Conclusion

To conclude, this study provided evidence for the first time that minority stressors have indirect effects on both internalizing and externalizing mental health outcomes through hopelessness. Thus, interventions targeting hopelessness should be implemented to ameliorate the adverse impacts of anti-trans stigma, while still considering the potential differences between different types of proximal stressors and between internalizing and externalizing mental health outcomes. The potential protective role of identity nondisclosure as a proximal minority stressor and the role of interpersonal processes on substance use should be addressed.

## Data Availability

The data that supports the findings include sensitive information and cannot be made publicly available. The data are available from the corresponding author, Banu Ünsal, upon reasonable request.

## Footnotes

1 Because this study aimed at understanding individuals’ experiences based on their self-identification and it was not concerned about biological/medical aspects of trans and non-binary experiences, we purposefully did not ask participants about their birth-assigned sex as suggested by several previous literature written by non-binary researchers themselves (e.g., Morgenroth & Ryan, 2021; Schudson & Morgenroth, 2022).

## Code Availability

The MPlus code that supports the findings is available from the corresponding author, Banu Ünsal, upon reasonable request.
